# Teaching Mindfulness to Teachers: a Systematic Review and Narrative Synthesis

**DOI:** 10.1007/s12671-017-0691-4

**Published:** 2017-02-23

**Authors:** Lisa-Marie Emerson, Anna Leyland, Kristian Hudson, Georgina Rowse, Pam Hanley, Siobhan Hugh-Jones

**Affiliations:** 1 Clinical Psychology Unit, Department of Psychology, University of Sheffield, Sheffield, S10 2TN UK; 2 School of Psychology, University of Leeds, Leeds, UK; 3 School of Education and Professional Development, University of Huddersfield, Huddersfield, UK

**Keywords:** Mindfulness, Teacher, Teacher stress, Self-efficacy, Interventions

## Abstract

School teachers report high levels of stress which impact on their engagement with pupils and effectiveness as a teacher. Early intervention or prevention approaches may support teachers to develop positive coping and reduce the experience and impact of stress. This article reviews research on one such approach: mindfulness-based interventions (MBIs) for school teachers. A systematic review and narrative synthesis were conducted for quantitative and qualitative studies that report the effects of MBIs for teachers of children aged 5–18 years on symptoms of stress and emotion regulation and self-efficacy. Twelve independent publications were identified meeting the inclusion criteria and these gave a total of 13 samples. Quality appraisal of the identified articles was carried out. The effect sizes and proportion of significant findings are reported for relevant outcomes. The quality of the literature varied, with main strengths in reporting study details, and weaknesses including sample size considerations. A range of MBIs were employed across the literature, ranging in contact hours and aims. MBIs showed strongest promise for intermediary effects on teacher emotion regulation. The results of the review are discussed in the context of a model of teacher stress. Teacher social and emotional competence has implications for pupil wellbeing through teacher–pupil relationships and effective management of the classroom. The implications for practice and research are considered.

## Introduction

Teaching is a highly stressful profession (Smith et al. 2000); approximately 40% of teachers in the USA leave the profession within the first 5 years of qualifying (Ingersoll 2002) and 73% of newly qualified teachers in the UK consider leaving (Association of Teachers and Lecturers 2015). Teachers report that current teaching climates generate high levels of stress, which lead to work-related fatigue, depression and anxiety, cynicism and low self-efficacy (NASUWT 2013). Furthermore, teacher stress and burnout (exhaustion with depressive symptoms; Swider and Zimmerman 2010) can negatively impact pupil engagement and learning through teacher absenteeism, reduced self-efficacy and diminished teaching effectiveness (for a review of the evidence, see Bricheno et al. 2009; Roeser et al. 2012). Given these potential negative and costly effects of teacher stress, there is impetus to identify effective interventions to support teachers to stay healthy and remain within the profession.

In the current paper, the term stress refers to physical symptoms, such as sleep disturbance, and psychological symptoms, such as depression, anxiety and burnout. Jennings and Greenberg (2009) argued that teachers’ capacity to cope with work-related stress relates to their social and emotional competence (SEC), defined as an awareness and ability to regulate emotions. In turn, emotion regulation facilitates teachers’ sense of mastery and protects wellbeing. Existing research on stress, emotion regulation and self-efficacy in teachers supports the proposal that these three constructs contribute to effective teaching practice.

Emotion regulation is the capacity to effectively manage one’s emotional reactivity, including the conscious and unconscious use of strategies, and internal or external resources, to decrease, maintain or increase positive and negative emotions, in either an anticipatory or responsive manner (Cole et al. 2004; Gross and Thompson 2007). Poor emotion regulation in teachers has been associated with more frequent and enduring negative affect, increased negative interactions with pupils, stress and burnout, and attrition from the profession (Darling-Hammond 2001; Montgomery and Rupp 2005). Specifically, two components of emotion regulation (poor emotional appraisal and self-regulation) significantly predicted burnout (Chan 2006), with effective emotion regulation predicting increased teacher self-efficacy (Chan 2004). Teacher self-efficacy is considered to be a protective factor against the effects of stress and burnout (Beltman, Mansfield, & Price 2011; Caprara et al. 2006). Self-efficacy is the belief in one’s ability to persevere with a course of action in pursuit of a valued goal (Bandura 1992). In the context of teaching, higher teacher self-efficacy has been linked to perseverance with challenging students and improved pupil behavior in the classroom (Robertson and Dunsmuir 2013), whilst lower teacher self-efficacy has been associated with increased stress and lower occupational commitment (Klassen and Chiu 2011).

Individual differences in emotion regulation and self-efficacy may contribute to a vulnerability to stress in teachers; therefore, interventions that target these constructs may protect teachers against potential stress. Mindfulness-based interventions have burgeoning promise for their applicability to occupational contexts, and there is emerging evidence for their efficacy in enhancing emotion regulation and self-efficacy. Mindfulness is the ability to focus attention on the present moment while possessing an orientation of openness, curiosity and non-judgement (Bishop et al. 2004). Meta-analytic reviews have reported medium to large effect sizes of MBIs on indicators of psychological health (Carmody and Baer 2009; De Vibe et al. 2012). Similarly, when delivered across occupational settings, there have been reported positive effects of MBIs on stress and wellbeing (health care providers, Escuriex and Labbé 2011; health care professionals, Irving et al. 2009; working adults, Virgili 2013). The traditional format for delivery of a MBI is eight weekly 2.5-h sessions, which incorporate formal mindfulness practices (e.g. sitting and movement meditation, body scanning and mindful eating), experiential group discussion, psycho-education and home practices. The two most well-established programmes are mindfulness-based stress reduction (MBSR; Kabat-Zinn 1990), which was originally developed for hospital patients experiencing conditions that were difficult to treat with medical interventions, and mindfulness-based cognitive therapy (MBCT; Segal et al. 2002), which was originally developed to prevent relapse of recurring depression and includes specific components from cognitive-behavior therapy.

In recent years, MBIs have been adapted and applied in the teaching context. Jennings and Greenberg (2009) have proposed their logic model, which suggests that MBIs work directly to help teachers recognise (i.e. greater self-awareness) and regulate stress reactions (i.e. emotion regulation; Jennings and Greenberg 2009). Consistent with this model, we propose that the main outcome of MBIs for teachers is reduced stress, with intermediary increases in emotion regulation and self-efficacy. Increased mindfulness and self-compassion, resulting from the MBI, serve as the mechanisms of these effects. Previous research has demonstrated that MBIs can improve emotion regulation (Chiesa et al. 2013) through greater self-awareness and attentional capacity, giving the individual an enhanced ability to detect (through greater awareness of thoughts and bodily sensations), decentre from, accept and modulate emotion in real time (Garland et al. 2011). MBIs can also positively alter one’s perspective on the self, specifically through increased decentering, reduced self-referential thoughts and increased self-compassion (Hölzel et al. 2011). Moreover, enhanced self-compassion can reduce negative appraisals of an individual’s teaching competence and thus increase self-efficacy (Neff 2003). Similarly, greater self-compassion acts as an effective emotion regulation strategy by responding to negative affective states (e.g. perceived inadequacy or failures as a teacher) with kindness rather than criticism (Hölzel et al. 2011). As such, research relating to MBIs often includes the measurement of trait mindfulness and self-compassion as indicators of cognitive changes that may precipitate other broader changes (e.g. decreased stress).

This systematic review aims to synthesise the current research evidence examining the effectiveness of MBIs for (i) reducing teacher stress as a main outcome, (ii) supporting gains in emotion regulation and self-efficacy as intermediary effects and (iii) mindfulness and self-compassion as mechanisms of action. The review includes a systematic search of published intervention studies where teachers of children aged 5–18 years have participated in an MBI. We will present a narrative review of the literature and discuss the results in the context of theoretical models of teacher stress. The limitations of the current evidence base and future directions for applying mindfulness in educational contexts will be considered.

## Method

### Search Strategy

A literature search was conducted in September 2015 (from 1966 onwards), across three electronic databases (PsycINFO, Web of Science, ERIC). Search terms were combined (‘AND’) across two key concept areas: (1) *mindfulness intervention* (‘mindfulness’, ‘MBSR’, ‘MBCT’, ‘meditation’) and (2) *teachers* (‘teacher’, ‘educator’, ‘school’). Hand searching reference lists and citations of relevant reviews (Albrecht, Albrecht and Cohen 2012; Tilahun and Vezzuto 2014) and backward and forward citation searches of identified articles completed the search strategy.

### Selection of Studies

Included articles delivered a MBI to teachers (qualified or trainee) of children aged 5–18 years, in either mainstream or special provision education (e.g. children with special needs), and assessed an aspect of teacher stress, self-efficacy or emotion regulation. Interventions with teachers in higher education or pre-school were excluded. Due to the relative infancy of this research area, a broad inclusion criterion was applied with regard to MBIs, and no specific criterion regarding the structure of the intervention was applied. However, studies were limited to approaches that drew upon established models of mindfulness (e.g. MBSR, MBCT) and explicitly integrated core experiential mindfulness practices (e.g. bodyscan, mindfulness of breath) during taught sessions and as home practice. Studies were included where the MBI was delivered across a number of sessions and included home practice content; studies of a single mindfulness session/lecture or practice were excluded. Interventions including large components of related therapies (e.g. Acceptance and Commitment Therapy, yoga or those where the meditation intervention was not explicitly labelled as mindfulness) were excluded.

Broad methodology criteria were applied. Quantitative, qualitative and mixed-methods designs were eligible for inclusion. Valid measures of teacher stress (psychological and physical symptoms, and burnout), self-efficacy and emotion regulation were included; other teacher measures and measures relating to classroom functioning or child behaviour were excluded. Included papers were written in English and published in peer-reviewed journals or awaiting publication.

Database searches returned 607 papers and manual searches returned 18. With duplicates removed, 600 titles and abstracts were screened. Seventeen papers were identified as suitable for full-text screening; five were excluded for not meeting the inclusion criteria. Twelve papers are included in the synthesis.

### Quality Appraisal

The quality of each paper was assessed independently by two researchers using the Quality Assessment Tool for Studies with Diverse Deigns (QATSDD; Sirriyeh et al. 2012). Up to 16 items (14 for quantitative, 14 for qualitative, 16 for mixed methods) were scored between 0 and 3 (0 = not at all, 1 = very slightly, 2 = moderately, 3 = completely), and the sum of these provides an overall score for the body of evidence which is expressed as a percentage of the maximum possible score for each study assessed. For the purposes of this review, an additional item assessed the extent to which the MBI was reported in sufficient detail (‘clarity of intervention’; 0–3); ‘sufficient’ was indicated by detail on core intervention components, dosage, method of delivery, by whom and in what context. Initial agreement between the two researchers was 91.6%, calculated using Cohen’s kappa (Cohen 1988). Discrepancies were resolved through discussion.

### Data Extraction

The following data were recorded for each qualifying study: publication details (e.g. author, country of study, year of publication), design (e.g. conditions, outcomes, randomisation, blinding, control group details), MBI details (e.g. duration, programme design) and population details (e.g. age, sample size, gender, years qualified). In addition, relevant data were extracted across four areas: i.e. (i) teacher stress, (ii) self-efficacy, (iii) emotion regulation and (iv) mindfulness and compassion. Data were extracted where it pertained to teachers and not when teacher data for teachers was combined with parent data.

### Effect Size Calculation

For consistency, effect sizes (ES; Cohen’s *d*) were computed from the published data for quantitative studies. Where insufficient data were reported in the published article, additional data were obtained from the authors (Jennings et al. 2011, study 2). Cohen’s *d* was calculated by extracting the mean difference and standard deviations from intervention and control groups (where appropriate; for formulae, see Higgins and Green 2011; Morris and DeShon 2002; Schmidt and Hunter 2014). Accepted categories (Cohen 1988) of small (0.2), medium (0.5) and large (0.8) effect sizes were applied. Due to the small number of studies and heterogeneity between designs, a meta-analysis was not conducted. Instead, the percentage of significant findings and the strength of the effect sizes are considered within each outcome section.

## Results

Twelve publications reported findings from 13 studies (two studies in Jennings et al. 2011). Table 1 provides key information regarding each publication. Data were reported from a total of 589 participants, with the majority from qualified teachers in mainstream educational settings. Most research was conducted in the USA and a minority in Canada and the UK. Quantitative designs dominated, including controlled and non-controlled studies, with a minority reporting qualitative data. The quality assessment of the literature suggested that strengths lie in the reporting of study details, including aims, recruitment and data collection procedures. In addition, there was generally a good fit between the research questions and methods employed to assess these. Studies that faired better in quality included a theoretical framework, had a reasonable sample size and detailed the psychometric properties of the tools and analytical methods used. On the whole, the body of literature was weakened by little consideration of sample size in terms of analysis, or the reliability of the analytical process (with the exception of Taylor et al. 2016). In addition, only studies by Jennings et al. (2011, 2013) included user involvement (i.e. feedback from teachers on the intervention) in the design.

**Table 1:** Details of reviewed studies

Author (year)	Country	*n*	Attrition %	Teacher % female	Teacher age (SD)	School level	SEN or MAIN	MBI	Design	Control group	Randomised	Quality rating %
Benn et al. (2012)	USA	38	7.9	91.4	45.6	Various	SEN	SMART-in-Education	Quan —independent groups, pre-post test, follow-up	Wait list	Yes	81%
Beshai et al. (2016)	UK	89	17.6	69.7%	–	Secondary	MAIN	Adapted MBSR/MBCT	Quan—independent groups, pre-post test	Wait list	No	79%
Flook et al. (2013)	USA	19	5.3	88.9	43.1 (9.87)	Elementary	MAIN	mMBSR	Quan—independent groups, pre-post test	Wait list	Yes	78%
Frank et al. (2015)	USA	36	–	77.8	40.72 (10.77)	High school	MIXED	Adapted MBSR	Quan—independent groups, pre-post test	Non-active	No	58%
Gold et al. (2010)	UK	11	9.1	–	–	Primary	MAIN	Closely followed MBSR	Quan—single group, pre-post	None	No	57%
Jennings et al. (2011) *study 1*	USA	31	6.5	93.6	40.0 (11.8)	Elementary	MAIN	CARE	Mixed—single group, pre-post-test and focus groups	None	No	71%
Jennings et al. (2011) *study 2*	USA	43	9.3	97.4	21.0 (5) trainee teachers 43.0 (12) mentors	Trainee teachers	MAIN	CARE	Mixed—independent groups, pre-post-test; classroom observations and focus groups	Wait list	Yes	71%
Jennings et al. (2013)	USA	53	5.6	88.7	36.0	Various	MAIN	CARE	Quan—independent groups, pre-post test	Wait list	Yes	91%
Napoli (2004)	USA	3	0.0	–	–	Elementary	MAIN	MBSR	Qual—interviews	–	–	41%
Poulin et al. (2008) *study 2*	Canada	44	–	73.0	26.4 (3.8)	Trainee teachers	MAIN	MBWE	Quan—independent groups, pre-post test	Non-active	No	50%
Roeser et al. (2013)	Canada/USA	113	7.1	88.5	46.9 (9.2)	Various	MAIN	SMART-in-Education	Quan—independent groups, pre-post-test, follow-up	Wait list	Yes	87%
Schussler et al. (2016)	USA	50 (44 teachers)	–	–	36 (22–60)	Various inc. elementary and secondary	MIXED	CARE	Qual—focus groups	–	–	76%
Taylor et al. (2016)	Canada	59	3.4	89.8	47 (28–63)	Elementary and secondary	MAIN	SMART	Mixed—independent groupspre/post/follow-up and survey	Wait list	Yes	72%

*MAIN* mainstream education setting, *SEN* Special Education Needs setting, *CARE* Cultivating Awareness and Resilience in Education, *MBSR* mindfulness-based stress reduction, *MBWE* mindfulness-based wellness education, *SMART* Stress Management and Relaxation Training in Education

### Mindfulness Programmes

Eight-week programmes based on MBSR and/or MBCT were examined in six studies (Beshai et al. 2016; Flook et al. 2013; Frank et al. 2015; Gold et al. 2010; Napoli 2004; Poulin et al. 2008). Adaptations for teachers were minimal and included greater reference to teaching practice and ways to bring mindful practices into the classroom, shorter sessions (75–120 min rather than 180 min standard) and shorter home practices (10–30 min rather than 45 min per day). Modified MBSR programmes included the Stress Management and Relaxation Training in Education (SMART), utilised in three studies (Benn et al. 2012; Roeser et al. 2013; Taylor et al. 2016). SMART has 70% overlap with MBSR, with additional content on emotion regulation, compassion and the application of mindfulness to teaching. SMART was delivered during 36 h contact time spread over 5–9 weeks. Cultivating Awareness and Resilience in Education (CARE) is a mindfulness-based programme specifically designed to promote teacher wellbeing, motivation and efficacy. CARE was utilised in four studies (Jennings et al. 2011 studies 1 and 2; 2013; Schussler et al. 2016); four daylong sessions were delivered over 4–5 weeks.

### Measures

Across the quantitative studies, 22 relevant measures assessed the three target areas of teacher functioning: stress (*n =* 16), emotion regulation (*n =* 4) and self-efficacy (*n =* 2). In addition, mindfulness was measured in eight studies, using one of two measures: the Five Facet Mindfulness Questionnaire (*n* = 7; FFMQ) or the original form, the Kentucky Inventory of Mindfulness Skills (*n* = 1; KIMS). Data for teachers’ self-compassion was available for three studies (Beshai et al. 2016; Flook et al. 2013; Roeser et al. 2013) using a version of the Self-compassion Scale (SCS, Neff 2003).

### Effects of MBIs

#### Stress

Forty-nine effect sizes, ranging from 0.01 to 2.12, were generated on the effects of a MBI on teacher stress, including psychological and physical symptoms, perceived stress and/or burnout (see Table 2).

**Table 2:** Symptoms of stress: calculated effect sizes from unadjusted means

Author (year)	Dependent variable/s	Effect sizes	Main findings	ES notes
Benn et al. (2012)	PSS STAI CES-D PANAS Neg Pos	−0.37 −0.50 −0.42 −0.52 0.11	Comparisons of pre- to post-intervention demonstrated significant intervention effects on anxiety and depression (*p* < 0.05), and nearing significance for stress (*p* < 0.10). Intervention effects on negative affect became significant at 2-month follow-up (*F* = 5.11, *p* < 0.05)	*d*_IGPP_ (SD_pre_)
Beshai et al. (2016)	PSS WEMWBS	−1.23 1.19	Significant reduction in stress (PSS: *t*(48) = 6.32, *p* < 0.001) and increase in wellbeing (WEMWBS: *t*(44) = −6.17, *p* < 0.001) from pre- to post- for intervention group only	*d*_IGPP_ (SD_pre_)
Flook et al. (2013)	SCL 90-R - GSI MBI EE Dep Pers	−0.08 −0.88 1.10 0.87	Significant decrease in symptoms (SCL GSI: *t*(9) = −3.66, *p* = 0.005) and burnout (MBI EE: *t*(9) = −2.42, *p* = 0.038; MBI Pers: *t*(9) = 3.03, *p* = 0.014) for intervention group Marginally significant increase in burnout for control group (MBI Pers: *t*(7) = −2.35, *p* = 0.051)	*d*_IGPP_ (SD_pre_)
Frank et al. (2015)	BSI - GSI Som Dep Anx MBI EE Dep Pers PSQI	−0.29 −0.24 −0.23 −0.30 −0.15 0.09 0.46 −2.12	No significant changes in symptoms (BSI) or burnout (MBI). Significant intervention effects indicated improvement in total sleep quality scores significantly for intervention group (*t*(29) = −4.21, *p* = 0.01, *d* = −1.53)	*d*_IGPP_ (SD_pre_)
Gold et al. (2010)	DASS -Dep Anx Stress	−0.93 −0.37 −0.70	Significant improvements in depression and stress symptoms (DASS Dep: *p* = 0.02; DASS stress: *p* = 0.05)	*d*_IGPP_ (SD_pre_)
Jennings et al. (2011) *study 1*	CES-D DPS TUS Task Gen PANAS Neg Pos	0.20 0.01 0.49 0.33 0.22 0.16	Significant improvement in time pressure (TUS Task: *p* = 0.01). Nearing significant improvement in (TUS Gen: *p* = 0.08)	*d*_RM_ (SD_D_)
Jennings et al. (2011) *study 2*	CES-D TUS Task Gen PANAS Neg Pos	−0.66 0.08 −0.08 −0.38 0.34	No significant differences reported.	*d*_IGPP_ (SD_pre_)
Jennings et al. (2013)	CES-D DPS TUS Task Gen MBI EE Dep Pers PANAS Neg Pos	−0.40 −0.77 −0.49 −0.42 0.02 0.19 0.40 −0.24 0.21	Significant intervention effects on physical symptoms (DPS: *F*(1, 47) = 10.2, *p* = 0.002), time pressure (TUS Gen: *F*(1, 47) = 5.4, *p* = 0.025) and personal accomplishment subscale of MBI (*F*(1, 47) = 3.9, *p* = 0.05)	*d*_IGPP_ (SD_pre_)
Poulin et al. (2008)	K10 SWLS	−0.64 0.59	Intervention effects observed for satisfaction with life (*F* = 6.56, *p* < 0.05)	*d*_IGPP_ (SD_pre_)
Roeser et al. (2013)	STAI BDI MBI total	−0.38 −0.37 −0.22	Significant intervention effects confirmed that intervention group reported fewer symptoms of anxiety (*F*(1, 53) = 7.11, *p* < 0.01) and depression (*F*(1, 53) = 10.67, *p* < 0.01) post-test for US sample only (maintained at 3-month follow-up) Significant intervention effects confirmed that intervention group reported less burnout post-test than control group (*F*(1, 108) = 14.96, *p* < 0.01; maintained at 3-month follow-up)	*d*_IGPP_ (SD_pre_)
Taylor et al. (2016)	Occupat. stress T1–T2 T2–T3	−0.32 0.24	Significant intervention effects confirmed that intervention group reported fewer symptoms of occupational job stress compared to those in the control condition at T2 (MMBI = 2.97, SD = 0.59 vs. MWC = 3.61, SD = 0.80), *F*(1, 54 = 8.20 = *p* < 0.01) and greater stress reduction compared to controls over the prior 9 weeks (MMBI = 2.46, SD = 0.93 vs. MWC = 3.39, SD = 0.72), *F*(1, 54 = 17.51 = *p* < 0.01)	*d*_IGPP_ (SD_pre_)

*PSS* Perceived Stress Scale, *STAI* State Trait Anxiety Inventory, *CES-D* Center for Epidemiological Studies—Depression Scale, *PANAS* Positive and Negative Affect Scale, *Pos* positive subscale, *Neg* negative subscale, *WEMWBS* Warwick-Edinburgh Mental Well-being Scale, *SCL 90R* Symptom Checklist 90R, *MBI* Masloch Burnout Inventory, *EE* emotional exhaustion, *Dep* depersonalisation, *Pers* personal accomplishment, *BSI* Brief Symptom Inventory (GSI—General Symptom Index), *Som* somatisation, *Dep* depression, *Anx* anxiety, *PSQI* Pittsburgh Sleep Quality Index, *DASS* Depression, Anxiety and Stress Scale, *Dep* depression, *Anx* anxiety, *DPS* daily physical symptoms, *TUS* Time Urgency Scale, *Task* Task-Related Hurry, *Gen* General Hurry, *ED-6* Teacher Stress Scale, *K10* Kessler-10 Psychological Distress Scale, *SWLS* Satisfaction with Life Scale, *BDI* Beck Depression Inventory, *Occ-Stress* occupational stress

There was considerable variation in the effects of MBIs on symptoms of anxiety and depression across studies; 44% of reported effects were significant. The studies that reported small–medium and significant improvements in symptoms of anxiety (2/4 studies) and depression (2/7 studies) achieved high quality ratings (Benn et al. 2012; Roeser et al. 2013). Effects on more general measures of psychological symptoms were reported in three studies with contrasting results. Flook et al. (2013) reported a significant improvement in symptoms (Symptom Check List, Derogatis 1994) in their intervention group, whereas Frank et al. (2015) reported no significant improvement. However, both studies report within-group comparisons and did not provide a comparison with the control group. Furthermore, the effect size reported by Flook et al. (2013) was calculated on post-intervention scores only (*d* = 0.53); therefore, the much smaller effect size calculated within this review might be a more accurate comparison of change between groups. Poulin et al. (2008) utilised control group comparisons and reported no improvements in overall psychological distress. The effects of MBIs on measures of general wellbeing were reported in six studies, with two reporting positive effects (Beshai et al. 2016; Poulin et al. 2008). No further immediate effects were observed on general wellbeing measures; however, Benn et al. (2012) reported improvement in negative affect, which became significant at 2-month follow-up.

The effects on both general and occupation-specific stress were reported in eight studies, with 60% of the results being significant. There was some variation in the quality of the studies reporting stress, with both low- (Gold et al. 2010) and high-quality (Jennings et al. 2013) studies reporting significant effects of mindfulness on stress. Improvements in occupation-specific stress were significant in three of the four studies reporting these findings; however, effect sizes were small and quality varied between the studies (Jennings et al. 2011—study 1; Jennings et al. 2013). Physical symptoms of stress were assessed in three studies, utilising three different measures. Two studies (Jennings et al. 2011 study 1; Jennings et al. 2013) utilised the Daily Physical Symptoms (DPS; Larsen and Kasimatis 1997) measure. In the lower-quality non-controlled study, no improvement was reported following mindfulness training (Jennings et al. 2011—study 1); however, the higher-quality randomised-controlled study, Jennings et al. (2013; rated excellent), reported a medium and significant improvement compared to the control group. Frank et al. (2015) reported small and non-significant intervention effects on somatisation, but large and significant improvements in sleep quality.

Four studies assessed burnout; 50% of results across three studies demonstrated significant improvements post-intervention. Specific improvements in ‘emotional exhaustion’ and ‘personal accomplishment’ were reported for the intervention group by Flook et al. (2013) and from between-group comparisons by Jennings et al. (2013). Roeser et al. (2013) reported a significant intervention effect in reducing burnout for those trained in mindfulness compared to controls, but this study received the lowest quality rating score out of the three studies.

#### Emotion Regulation

Four quantitative studies reported the impact of MBIs for teachers on measures of emotion regulation (Table 3). Eight effect sizes were generated, ranging from 0.43 to 1.56. Significant positive effects of MBIs on emotion regulation were reported for 63% of the results.

**Table 3:** Teacher emotion regulation: calculated effect sizes from unadjusted means

Author (year)	Dependent variable/s	Effect sizes	Main findings	ES notes
Benn et al. (2012)	ERWSES	0.43	Intervention effects approached significance (*p* < 0.10)	*d*_IGPP_ (SD_pre_)
Frank et al. (2015)	ASRES Acknow Calm Pres Mom Accept	1.24 1.56 1.16 0.46	Significant differences between intervention and control groups on change scores (post-pre) indicated intervention effect for self-efficacy in acknowledgement (*t*(33) = 3.71, *p* = 0.03, *d* = 1.25, calmness, *t*(33) = 4.36, *p* = 0.02, *d* = 1.47) and present moment (*t* (33) = 3.69, *p* = 0.01, *d* = 1.25). No significant improvements were found for the measure of efficacy in acceptance (*t* (33) = 1.10, *p* = 0.40, *d* = 0.37)	*d*_IGPP_ (SD_pre_)
Jennings et al. (2013)	ERQ Reapp Supp	−0.99 −0.57	Significant intervention effects on emotion regulation (ERQ Reapp: *F*(1,47) = 10.9, *p* = 0.002)	*d*_IGPP_ (SD_pre_)
Taylor et al., (2016)	Emotional Reg. efficacy	0.50	Significant intervention effects for efficacy for regulating emotions (MMBI = 3.37, SD = 0.60 vs. MWC = 3.00, SD = 0.85), *F*(1, 54 = 7.06 = *p* < 0.05)	*d*_IGPP_ (SD_pre_)

*ERWSES* Emotion Regulation at Work Self-efficacy Scale, *ASRES* Affective Self-regulatory Efficacy Scale, *Acknow* acknowledgement, *Pres Mom* present moment, *Accept* acceptance, *ERQ* Emotion Regulation Questionnaire

Frank et al. (2015) reported significant changes from within-group analyses for the intervention group on subscales ‘acknowledgement’, ‘remaining calm’, and ‘present moment focus’; the authors hypothesised that these specific areas of self-regulation were most likely to improve during the intervention. However, in two studies of superior quality, which directly compared intervention and control group changes, mixed findings were reported. Benn et al. (2012) reported no significant change on a teaching-specific self-regulation measure (Emotion Regulation at Work Self-efficacy Scale, ERWSES), although the medium effect did approach significance. In comparison, Jennings et al. (2013) reported a large and significant effect on one of two subscales on a general measure of emotion regulation (Emotion Regulation Questionnaire, ERQ; Gross and John 2003); changes on the ‘reappraisal’ subscale reflected teachers’ ability to regulate their emotions and consequently reappraise stressful situations in the context of teaching students. The differences in results may have been due to the difference in outcome measures. The two studies that reported significant findings (Frank et al.; Jennings et al.) utilised a measure of general emotion regulation, whereas no significant changes were reported for the teacher-specific ERWSES utilised by Benn et al. There appears to be a strong effect of mindfulness on emotion regulation more generally, and a possible weaker effect on teaching-specific emotion regulation, which is designed to assess emotion. A larger sample size may have made this effect detectable.

Findings from the two qualitative studies were consistent with the quantitative data, suggesting improvements in emotion regulation following a MBI. Teachers in the Schussler et al. (2016) study reported increased emotional awareness and reduced reactivity in emotional situations; the breathing and emotion awareness exercises learned as part of the MBI were commonly utilised by teachers to effect this outcome. Similarly, in the Taylor et al. (2016) study, teachers reported increased confidence in coping with negative emotions in the workplace; the authors suggested that increased emotion regulation efficacy may serve as a potential mediator in stress reduction (evidenced by their quantitative findings).

#### Self-efficacy

The effects of mindfulness training on teacher self-efficacy were measured in five studies (Table 4). Effect sizes ranged from 0.07 to 0.87. Significant benefits of mindfulness training on outcomes were reported for 29% of the results.

**Table 4:** Teacher self-efficacy: calculated effect sizes from unadjusted means

Author (year)	Dependent variable/s	Effect sizes	Main findings	ES notes
Benn et al. (2012)	10 items taken from Midgley et al. (2000)	0.65	No significant intervention effects reported	*d*_IGPP_ (SD_pre_)
Jennings et al. (2011) *study 1*	TSES Student Instruction Class mgt	0.07 0.30 0.18	No significant effects reported	*d*_RM_ (SD_D_)
Jennings et al. (2011) *study 2*	TSES Student Instruction Class mgt	0.50 0.56 0.17	No significant differences observed between intervention and control groups at post-test	*d*_IGPP_ (SD_pre_)
Jennings et al. (2013)	TSES Student Instruction Class mgt	0.51 0.59 0.31	Significant intervention effects improved teacher efficacy, specifically in student engagement (TSES student: *F*(1, 47) = 10.3, *p* = 0.002) and instruction (TSES instruction: *F*(1, 47) = 11.6, *p* = 0.001)	*d*_IGPP_ (SD_pre_)
Poulin et al. (2008)	TSES total Student Instruction Class mgt	0.78 0.87 0.53 0.51	Significant intervention effect on overall self-efficacy (*F* = 4.88, *p* < 0.05); intervention group showed greater improvement in self-efficacy, student engagement than control group (*F* = 4.51, *p* < 0.05)	*d*_IGPP_ (SD_pre_)

*TSES* Teachers’ Sense of Efficacy Questionnaire, *Student* student engagement, *Instruction* instructional practices, *Class Mgt* classroom management

Four of the five studies utilised the Teachers’ Sense of Efficacy Questionnaire (TSES; Tschannen-Moran and Woolfolk Hoy 2001); two reported medium–large effects and significant improvements on the ‘student engagement’ subscale (Jennings et al. 2013; Poulin et al. 2008), and one reported an additional medium effect and significant change on the ‘instruction’ subscale (Jennings et al. 2013). These findings were not replicated across the two other studies (Jennings et al. 2011 study 1 and 2); however, these studies did not make change score comparisons between intervention and control groups. No significant effects on the ‘classroom management’ subscale of the TSES were reported. Benn et al. (2012) utilised a measure of teaching self-efficacy designed for the purposes of their study; their reported medium effect size was not significant.

Inconsistent with positive quantitative effects on efficacy, the qualitative data reported by Schussler et al. (2016) did not include any description of changes in work-related efficacy.

#### Mindfulness and Compassion

All quantitative studies reported the influence of MBIs for teachers on measures of mindfulness and self-compassion (Table 5). Effect sizes ranged from 0.04 to 1.77. Significant benefits of mindfulness training on outcomes were reported for 39% of the results.

**Table 5:** Teacher mindfulness and self-compassion: calculated effect sizes from unadjusted means

Author (year)	Dependent variable/s	Effect sizes	Main findings	ES notes
Beshai et al. (2016)	FFMQ SCS	1.45 1.06	Significant increase in mindfulness (FFMQ: *t*(48) = −9.31, *p* < 0.001) from pre- to post- for intervention group only Significant increase in self-compassion from pre- to post-intervention for both groups (*F*(1,87) = 18.90, *p* < 0.001)	*d*_IGPP_ (SD_pre_)
Flook et al. (2013)	FFMQ Observe Describe Act Non-judge Non-react SCS Humanity	0.24 0.55 0.20 −0.04 0.16 0.80	Significant increase in mindfulness (describe subscale: *t*(9) = 2.53, *p* = 0.032) and self-compassion (SCS humanity: *t*(9) = 3.42, *p* = 0.008) in intervention group. No changes in control group Correlations between change scores demonstrated significant association between mindfulness and improvements in symptoms (SCL GSI with FFMQ acting with awareness: *r* = −0.76, *p* = 0.010; SCL GSI with FFMQ nonreactivity: *r* = −0.78, *p* = 0.007) and burnout (MBI EE with FFMQ acting with awareness: *r* = −0.70, *p* = 0.024; MBI Dep with FFMQ nonreactivity: *r* = −0.80, *p* = 0.006) in the intervention group, but no significant correlations in the control group	*d*_IGPP_ (SD_pre_)
Frank et al. (2015)	FFMQ Observe Describe Act Non-judge Non-react	1.12 0.65 1.11 0.58 1.77	Significant intervention effects for observe (*t*(23) = 4.63), act with awareness (*t*(23) = 2.66, *p* = 0.03, *d* = 1.06), non-judgment (*t*(23) = 3.76, *p* = 0.01, *d* = 1.50) and non-reactivity (*t*(23) = 3.95, *p* = 0.01, *d* = 1.58).	*d*_IGPP_ (SD_pre_)
Jennings et al. (2011) *study 1*	FFMQ Observe Describe Act Non-judge Non-react	0.65 0.65 0.30 0.35 0.65	Significant improvements in mindfulness, specifically observing (*p* < 0.01), describing (*p* < 0.01) and non-reactivity (*p* < 0.01). Close to significant effects on acting with awareness (*p* = 0.10) and non-judging (*p* = 0.06)	*d*_RM_ (SD_D_)
Jennings et al. (2011) *study 2*	FFMQ Observe Describe Act Non-judge Non-react	0.63 0.17 −0.28 0.14 0.20	No significant differences between intervention and control groups post-test	*d*_IGPP_ (SD_pre_)
Jennings et al. (2013)	FFMQ total Observe Describe Act Non-judge Non-react	0.38 0.74 0.36 −0.28 −0.10 0.68	Significant intervention effects on mindfulness: overall mindfulness score (*F*(1, 47) = 4.29, *p* = 0.044) and subscales: observing (*F*(1, 47) = 9.8, *p* = 0.003) and non-reactivity (*F*(1, 47) = 8.4, *p* = 0.006).	*d*_IGPP_ (SD_pre_)
Poulin et al. (2008)	KIMS total Observe Describe Act Non-judge	0.78 0.57 0.21 0.64 0.35	Significant intervention effects on overall mindfulness (KIMStotal: *F* = 12.56, *p* < 0.001); intervention group improved significantly more than control group on the observe (*F* = 8.03, *p* < 0.01) and act with awareness subscales (*F* = 13.52, *p* < 0.01)	*d*_IGPP_ (SD_pre_)
Roeser et al. (2013)	FFMQ total Occupational SCS	0.52 0.48	Intervention effects reported for mindfulness (*F*(1, 109) = 16.92, *p* < 0.01) and occupational self-compassion (*F*(1, 107) = 31.14, *p* < 0.01) confirmed greater improvements for intervention group compared to control group (both maintained at 3-month follow-up)	*d*_IGPP_ (SD_pre_)

*FFMQ* Five Facet Mindfulness Questionnaire, *KIMS* Kentucky Inventory of Mindfulness Skills, *SCS* Self-compassion scale, *Occ-SCS* Occupational Self-Compassion Scales

Total mindfulness scores were reported and improved across three studies with control group comparisons, with small–medium effect sizes (Jennings et al. 2013; Poulin et al. 2008; Roeser et al. 2013). The majority of studies conducted subscale analysis and consistently reported medium–large and significant effects on the ‘observe’ (Flook et al. 2013; Frank et al. 2015; Jennings et al. 2011—study 1; Jennings et al. 2013; Poulin et al. 2008) and ‘non-reactivity’ subscales (Frank et al. 2015; Jennings et al. 2011—study 1; Jennings et al. 2013). Further significant changes were reported for medium effects on ‘describe’ (Flook et al. 2013; Jennings et al. 2011—study 1) and medium–large effects on ‘act with awareness’ (Frank et al. 2015; Poulin et al. 2008). Although findings were not conclusive across the studies, there was some promising evidence that the ability of teachers to be mindful increased post-MBI.

Three studies assessed self-compassion. Beshai et al. (2016) and Flook et al. (2013) reported large and significant effects on self-compassion. Roeser et al. (2013) adapted the SCS to assess teachers’ tendency to have compassion toward themselves as teachers; a medium and significant change was reported for the intervention group compared to the control group.

Three studies reported qualitative data that indicated consistent changes in mindfulness, as described by participating teachers. Teachers attributed an increased awareness of their sensory input (Napoli 2004), bodily states and their physical and emotional health (Schussler et al. 2016) to the MBI. Furthermore, teachers consistently reported the use of mindfulness in their personal lives, including the use of practices to ameliorate stress (Napoli 2004; Schussler et al. 2016). Taylor et al. (2016) reported that teachers were more compassionate toward themselves, citing increased awareness of the need for self-care and permission to do this.

## Discussion

**Fig. 1:**
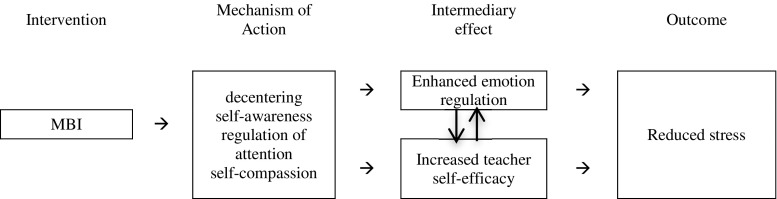
Model for proposed mechanisms of mindfulness-based interventions to reduce psychological distress

This review has examined the current evidence for the effects of MBIs on stress, emotion regulation and self-efficacy. The present review of evidence informs a proposed model of reduced teacher stress resulting from a MBI (Fig. 1). Through participation in mindfulness training, an individual may see gains in mindfulness (e.g. decentering, regulation of attention) and self-compassion (Hölzel et al. 2011) that lead to more effective emotion regulation strategies (Chiesa, Serretti, & Jakobsen 2013) and increased professional self-efficacy (Neff et al. 2005) and ultimately reduced stress (e.g. Carmody and Baer 2009; De Vibe et al. 2012). Importantly, within this model, there is a reciprocal interaction between increased self-efficacy and emotion regulation, as enhanced teacher self-efficacy increases effective emotion regulation and appraisal of effective regulation of affect (in a teaching context) enhances a sense of teaching efficacy (Bandura et al. 2003). Although the studies reviewed were not designed to directly test this model and as such the full model was not tested in this review, there is some support for gains in emotion regulation and reduced teacher stress following the MBIs. In addition, around a third of the increases shown in trait mindfulness and self-compassion were significant with variable effect sizes.

From the evidence reported across the 13 eligible studies, it is possible to conclude that MBIs for teachers show most promise for the proposed intermediary effect of emotion regulation. Effect sizes for emotion regulation tended to be larger with more of them showing statistical significance, although effects varied according to the measures used. Furthermore, qualitative data from teachers also demonstrated that the aspect of MBIs most commonly rated as helpful related to emotion awareness, recognition and understanding (Schussler et al. 2016; Taylor et al. 2016). Emotion regulation is proposed to be a key contributing factor toward teacher SEC (Jennings and Greenberg 2009); thus, the observed improvements in emotion regulation as a result of participating in a MBI would theoretically improve a teacher’s overall SEC. Jennings and Greenberg highlight the cascading benefits of improved teacher SEC for the classroom environment, and individual pupil wellbeing. However, none of the studies reviewed reported comparisons of emotion regulation at their chosen follow-up points (the longest of which was 3 months) making it difficult to ascertain lasting effects. Furthermore, there was a lack of data on the hypothesised cascading effects of mindful teachers in the classroom and on pupil outcomes. Long-term studies are therefore needed to assess changes in teacher effectiveness, classroom climate and pupil relationships.

Across studies, there was nascent promise for application of MBIs for reducing physical and psychological symptoms of stress, including burnout. Effect sizes were variable and the significance of results was inconsistent across studies. However, the greatest proportion of significant findings was for outcomes relating to teachers’ perceived stress. It may be that a distinction between perceived *stress* and subsequent *distress* (e.g. anxiety, depression, burnout) is necessary when studying the effects of MBIs in occupational settings. MBIs in occupational settings may be more fruitful in reducing stress, and thereby possibly preventing subsequent distress, rather than reduced current symptoms of distress. This proposal is consistent with the largest effects on emotion regulation, as a possible intermediary effect in reducing or preventing stress in the longer term. Longer follow-up and prospective studies are required to confirm these proposed preventative effects.

The inconsistency in findings about the impact of MBIs on psychological outcomes across studies could be due to intervention as well as methodological differences. There was a heterogeneous approach to delivering MBIs across the studies reviewed. It is beyond the scope of this review, and the stage of the evidence, to provide a detailed consideration of the content and theoretical underpinnings between the MBIs employed across studies; however, it is important to note the heterogeneity in terms of aims, content, duration and mode of delivery. The SMART-in-Education and CARE programmes include specific instruction on emotion management skills. Therefore, it is difficult to conclude whether the significant effects observed in the studies utilising these programmes are due to the mindfulness components of the course or taught elements on emotion regulation. It is possible that the effects of mindfulness training are enhanced when coupled with additional techniques, and the effectiveness of embedding mindfulness training alongside other stress reduction techniques for teachers merits investigation. Some studies reported that the mindfulness component of the intervention was adapted for teachers (Flook et al. 2013), whereas others were not (Gold et al. 2010). Based on current evidence, it is difficult to determine if tailoring for teachers promotes better outcomes. For example, the CARE intervention is highly tailored, yet did not consistently improve teacher self-efficacy across three studies (Jennings et al. 2011, 2013). In contrast, mindfulness-based wellness education (MBWE) has only minor adaptations for teachers and was associated with significant changes in self-efficacy (Poulin et al. 2008). These nuances in the design of the mindfulness programme are likely to have an impact on how effective the course is, and thus the outcomes reported. As the evidence base grows, it will be important to consider the effective components of MBIs for teachers.

Quantitative and qualitative data reported across studies suggest an improvement in teachers’ ability to be mindful, which indicates successful manipulation effects of the MBIs. However, in line with the broader work on MBIs, there is a need to better understand and isolate the mechanisms of change associated with participation in these interventions (Garland et al. 2011; Holzel et al. 2011). For example, group support is known to reduce work-based stress (Michie and Williams 2003) and is likely to be an important feature of the MBI experience (e.g. Irving, et al. 2014). Future research should focus on separating the effects of group attendance and intervention-specific effects by comparison with active group controls.

Overall, the body of literature reviewed demonstrated a number of limitations that need to be addressed in future research, including small sample sizes, insufficient statistical power and the absence of active controls. Where MBIs have been compared to an active control intervention for health care professionals, no significant differences were observed between groups, with each reporting greater relaxation and life satisfaction (Poulin et al. 2008), demonstrating the importance of this experimental control. As can be the case in intervention studies, teachers in the reviewed studies were not blind to the intervention aim and were also self-selecting. This is routine in psychological interventions (and in MBIs) where the readiness for intervention, and the personal decision to engage, is deemed important to outcomes as well as reflecting ethical practice (Lyubomirsky et al. 2011; Seligman et al. 2006; Seligman et al. 2005). It is unknown whether referring teachers to MBIs, or delivering them wholesale to a school staff, would be an effective, preventative approach to stress management.

Finally, only three studies reported fidelity checks (Benn et al. 2012; Jennings et al. 2013; Roeser et al. 2013). Checks of fidelity can be helpful for ensuring that reported effects result from the application of specific interventions and not other extraneous variables (Horner et al. 2006).

### Limitations of Review

The search strategy utilised in the current review may have introduced bias in study selection. Namely, the search identified studies which were conducted in developed, English-speaking countries (mostly in the USA), which limits the robustness and generalisability of the conclusions. However, the small number of studies reflects the infancy of research into mindfulness for teachers. Research on mindfulness in school settings is sparse and has tended to focus on the implementation and effects of mindfulness with school pupils. There are a greater number of studies that have investigated the occupation-related effects of MBIs for health professions with positive results (e.g. Escuriex and Labbé 2011).

As the search was based on electronic sources, grey/unpublished literature was not included, which may have resulted in some relevant studies being missed. In addition, publication bias is left unknown. Publication bias refers to the reduced likelihood that small studies with low or opposite effects will be published, due to either non-submission for publication or rejection at the review stage, whilst small studies with very high effect sizes are more likely to be published. Although some reviews detect small publication biases (e.g. Eberth and Sedlmeier’s (2012) meta-analysis of mindfulness meditation vs MBSR), most major reviews of MBIs indicate that publication bias is unlikely to be having any meaningful impact on effect sizes (De Vibe et al. 2012; Khoury et al. 2013; Piet and Hougaard 2011). These observations, together with the current interest in mindfulness in education, make it unlikely that publication bias has affected our identified effect sizes.

### Recommendations for Research and Practice

There are a number of potential barriers to introducing mindfulness to teachers. Firstly, the potential participants and their senior leadership team (SLT) need to be confident that a MBI has good potential to be helpful to teachers. Currently, there is only limited evidence of its benefit to the profession for managing stress. Current implementation models in the UK include wholesale provision to staff, with an opt-in approach. However, it may be too exposing or uncomfortable for staff from any one school to talk about their workplace stress together, given that some stress originates from work relationships and leadership decisions. There may also be concerns that offering an individual-level approach to stress management removes responsibility from organisations to protect employees from stress via workload management. However, not everyone is equally resourced to manage normative stress, stress is not equally distributed, and not all stress is workload related. Thus, offering a MBI as a way to support teachers may be appropriate, alongside a continued responsibility of employers to support a healthy work environment for their staff. The cost of delivering MBIs may be prohibitive for some schools. Some councils in England are supporting the delivering of MBIs to schools for free, although these represent highly localised provision (e.g. https://www.blackpool.gov.uk/News/2014/July/500k-to-boost-resilience-in-Blackpools-young-people.aspx). Furthermore, whilst several studies have identified the cost-effectiveness of MBIs for health outcomes in clinical and non-clinical populations (e.g. Kuyken et al. 2015), we do not yet understand the return on investment in school settings.

Finally, the current lack of convincing evidence of the positive effects of teacher MBIs on teaching and pupil outcomes may reduce the attraction of MBIs to schools. School priorities are academic performance, and as yet, there have been no large-scale, rigorous studies that show a strong relationship between mindfulness training for either teachers or pupils and attainment outcomes. However, there is increasing interest in how mindfulness might be delivered as a whole school approach to support a school climate that is conducive to staff and pupil self-awareness, other-awareness and wellbeing, with a view that these changes would indirectly facilitate academic engagement and performance.

There is a need to grow the evidence base for the effectiveness of MBIs with teachers and more comprehensive explorations of where those effects can be identified, for whom and under what conditions. It would be useful for future studies to aim for a more systematic approach focusing on robust research designs to address the limitations identified in this review. In particular, we recommend that future studies utilise theoretical frameworks as a basis of design, and testing specific hypotheses regarding the effects of MBIs for teachers. For example, the model of teacher stress proposed herewith could be tested through the inclusion of measures of identified mechanisms of change (mindfulness, self-compassion) and intermediary effects (teacher self-efficacy, emotion regulation) on stress (self-reported stress, burnout). Future studies will ideally include randomised controlled trials with both active and ‘business-as-usual’ control groups, with longer-term follow-ups. Consensus on outcome measures, including objective measures (e.g. absences from work, retention in the profession), would aid comparison across studies and allow wider conclusions to be drawn about effectiveness. If the benefits of MBIs can be robustly demonstrated for teachers, the impact on their pupils should then be ascertained. Again, utilising a theoretical framework will provide direction in terms of hypothesised effects. Jennings and Greenberg’s model of the socio-emotional classroom highlights specific direct and indirect effects that can result from improved teacher wellbeing. These ultimately can result in measurable benefits to students’ social, emotional and academic outcomes via intermediary changes observable in the classroom, such as improved teacher–student relationships.
